# The computational prediction of drug-disease interactions using the dual-network L_2,1_-CMF method

**DOI:** 10.1186/s12859-018-2575-6

**Published:** 2019-01-05

**Authors:** Zhen Cui, Ying-Lian Gao, Jin-Xing Liu, Juan Wang, Junliang Shang, Ling-Yun Dai

**Affiliations:** 1 School of Information Science and Engineering, Qufu Normal University, Rizhao, 276826 China; 2 Library of Qufu Normal University, Qufu Normal University, Rizhao, China

**Keywords:** Drug-disease interactions, L_2,1_-norm, Gaussian interaction profile, Matrix factorization

## Abstract

**Background:**

Predicting drug-disease interactions (DDIs) is time-consuming and expensive. Improving the accuracy of prediction results is necessary, and it is crucial to develop a novel computing technology to predict new DDIs. The existing methods mostly use the construction of heterogeneous networks to predict new DDIs. However, the number of known interacting drug-disease pairs is small, so there will be many errors in this heterogeneous network that will interfere with the final results.

**Results:**

A novel method, known as the dual-network L_2,1_-collaborative matrix factorization, is proposed to predict novel DDIs. The Gaussian interaction profile kernels and L_2,1_-norm are introduced in our method to achieve better results than other advanced methods. The network similarities of drugs and diseases with their chemical and semantic similarities are combined in this method.

**Conclusions:**

Cross validation is used to evaluate our method, and simulation experiments are used to predict new interactions using two different datasets. Finally, our prediction accuracy is better than other existing methods. This proves that our method is feasible and effective.

## Background

On average, it takes over a dozen years and approximately 1.8 billion dollars to develop a drug [1]. In addition, most drugs have strong side effects or undesirable effects on patients, so these drugs cannot be placed on the market. Therefore, many pharmaceutical companies resort to repositioning of existing drugs on the market [2]. Many known drugs can be found to have new effects for different diseases. In medicine, drug repurposing has two advantages. One advantage is that known drugs have already been approved by the US FDA (Food and Drug Administration) [3]. In other words, these drugs are safe to use. Another advantage is that the side effects of these drugs are known to medical scientists, so these side effects can be better controlled to achieve the desired therapeutic effect. Drug repurposing can help accelerate and facilitate the research and development process in the drug discovery pipeline [4].

The most important factor for drug repositioning is online biological databases. Many public databases, such as KEGG [5], STITCH [6], OMIM [7], DrugBank [8] and ChEMBL [9] store large amounts of information related to drugs and diseases. These databases contain detailed information such as a drug’s chemical structure, side effects, and genomic sequences [10].

In general, the goal of drug repositioning is to discover novel drug-disease interactions (DDIs) using existing drugs. Because a drug is often not specific for one disease, most drugs can treat a variety of diseases. Recently, more methods have been proposed for drug repositioning, such as machine learning [11], text mining [12], network analysis [13] and many other effective methods due to the increasing depth of research [14, 15]. Of course, we can also use the opposition-based learning particle swarm optimization to predict interactions, such as SNP-SNP interactions [16]. For instance, Gottlieb et al. proposed a computational method to discover potential drug indications by constructing drug-drug and disease-disease similarity classification features [17]. Then, the predicted score of the novel DDIs can be calculated by a logistic regression classifier. Napolitano et al. calculated drug similarities using combined drug datasets [18]. They proposed a multi-class SVM (Support Vector Machine) classifier to predict some novel DDIs. Moreover, some researchers use network-based models for drug repositioning. The advantage of this network model is that it can fully consider the large-scale generation of high-throughput data to build complex biological information interaction networks. Wang et al. proposed a method called TL-HGBI to infer novel treatments for diseases [19]. These authors constructed a heterogeneous network and integrated datasets about drugs, diseases and drug targets. Another network-based prioritization method called DrugNet was proposed by Martinez et al. [20]. This method can predict not only novel drugs but also novel treatments for diseases. Similar to the TL-HGBI method, the DrugNet method uses a heterogeneous network to predict novel DDIs using information about drugs, diseases, and targets. Luo et al. developed a computational method to predict novel interactions of known drugs [21]. Furthermore, comprehensive similarity measures and Bi-Random Walk (MBiRW) algorithm have been applied to this method. In addition, Luo et al. continued to propose a drug repositioning recommendation system (DRRS) to predict new DDIs by integrating data sources for drugs and diseases [14]. A heterogeneous drug-disease interaction network can be constructed by integrating drug-drug, disease-disease and drug-disease networks. Moreover, a large drug-disease adjacency matrix can replace the heterogeneous network, including drug pairs, disease pairs, known drug-disease pairs, and unknown drug-disease pairs. A fast and favourable algorithm SVT (Singular Value Thresholding) [22] has been used to complete predicted scores of the drug-disease adjacency matrix for unknown drug-disease pairs. According to previous studies, each method has its own advantages for predicting DDIs. However, after comparing the prediction of these methods, the best method is currently DRRS. The method achieves the highest AUC (area under curve) value and the best prediction [14]. Recently, matrix factorization methods have also been used to identify novel DDIs [23]. The matrix factorization method takes one input matrix and attempts to obtain two other matrices, and then the two matrices are multiplied to approximate the input matrix [23]. Similar to looking for missing interactions in the input matrix, matrix factorization can be used as a good technique to solve the prediction problem. Examples of such matrix factorization methods are the kernel Bayesian matrix factorization method (KBMF2K) [24] and the collaborative matrix factorization method (CMF) [25].

In this work, a simple yet effective matrix factorization model called the Dual-Network L_2,1_-CMF (Dual-network L_2,1_-collaborative matrix factorization) is proposed to predict new DDIs based on existing DDIs. However, there are many missing unknown interactions, so a pre-processing step is used to solve this problem. The main purpose of this pre-processing method is to attempt to weight K nearest known neighbours (WKNKN) [26]. Specifically, in the original matrix, WKNKN is used to describe whether there is an interaction between drug-disease pairs, bringing each element closer simply 0 and 1 to a reliable value than. Thus, WKNKN will have a positive impact on the final prediction. Furthermore, unlike the previous matrix factorization methods, L_2,1_-norm [2] and GIP (Gaussian interaction profile) kernels are added to the CMF method. Among them, L_2,1_-norm can avoid over-fitting and eliminate some unattached disease pairs [27]. The GIP kernels are used to calculate the drug similarity matrix and the disease similarity matrix [28]. Cross validation is used to evaluate our experimental results. The final experimental results show that after removing some of the interactions, our proposed method is superior to other methods. In addition, a simulation experiment is conducted to predict new interactions.

The results are described in Section 2, including the datasets used in our study and experimental results. The corresponding discussions are presented in Section 3. The conclusion is described in Section 4. Finally, Section 5 describes our proposed method, including specific solution steps and iterative processes.

## Results

### DDIs datasets

Information about the drugs and diseases was obtained from Gottlieb et al. [17], and the Fdataset comprises multiple data sources. It is the gold standard dataset. This dataset includes 1933 DDIs, 593 drugs and 313 diseases in total. Further information about the drugs and diseases are obtained from Luo et al. [21], and the Cdataset comprises multiple data sources. The Cdataset includes 2353 DDIs, 663 drugs and 409 diseases, including drugs from the DrugBank database and diseases from OMIM (Online Mendelian Inheritance in Man) database [7].

Both datasets contain three matrices: **Y** ∈ ℝ^*n* × *m*^, **S**_D_ ∈ ℝ^*n* × *n*^ and **S**_d_ ∈ ℝ^*m* × *m*^. The adjacency matrix **Y** is proposed to describe the association between drug and disease. In the adjacency matrix, *n* drugs are represented in rows and *m* diseases are represented in columns. If drug *D*(*i*) is associated with disease *d*(*j*), the entity **Y**(*D*(*i*), *d*(*j*)) is 1; otherwise it is 0. Sparsity is defined as the ratio of the number of known DDIs to the number of all possible DDIs [14]. Table 1 lists the specific information for these two datasets.

**Table 1:** Drugs, Diseases, and Interactions in Each Dataset

Datasets	Drugs	Diseases	Interactions	Sparsity
Cdataset	663	409	2532	9.337 × 10^−3^
Fdataset	593	313	1933	1.041 × 10^− 2^

### Similarities in the chemical structures of the drugs

The drug similarity matrix is used to predict interactions. The chemical structure information of the drugs constitutes this matrix, **S**_D_. The similarity information is derived from the Chemical Development Kit (CDK) [29], and the drug-drug pairs are represented as their 2D chemical fingerprint scores.

### Similarities in disease semantics

The disease similarity matrix was used to predict interactions. The matrix **S**_d_ is represented by the medical descriptions of the diseases. The similarities between disease-disease pairs were obtained from MimMiner [30]. Therefore, the semantic similarities of the diseases is achieved through text mining. Finally, the meaningful medical information is selected and meaningless data is discarded.

### Cross validation experiments

In this study, our experiments are compared to the previous methods (KBMF, HGBI, DrugNet, MBiRW, and DRRS). For each method, 10-fold cross validation is repeated ten times. However, before running our method, the pre-processing steps is performed first. The purpose is to solve the problem of missing unknown interactions. This pre-processing step improves the accuracy of the prediction to some extent.

We observe that the interactions between drugs and diseases remain fixed during cross-validation. In general, the receiver operating characteristic (ROC) curve can be described by changing the true positive rate (TPR, sensitivity) of different levels of the false positive rate (FPR, 1-specificity). Moreover, sensitivity and specificity (SPEC) can be written as follows:

1 $$ Sensitivity=\frac{TP}{TP+ FN}, $$

2 $$ SPEC=\frac{TN}{N}=\frac{TN}{TN+ FP}, $$

where *N* represents the number of negative samples, *TP* represents the number of positive samples correctly classified by the classifier and *FP* represents the number of false positive samples classified by the classifier. Similarly, *TN* represents the number of negative samples correctly classified by the classifier, and *FN* represents the number of false negative samples.

A popular evaluation indicator AUC is used to evaluate our approach [31]. AUC is defined as the area under the ROC curve, and it is obvious that the value of this area will not be greater than 1. In general, the value of AUC ranges between 0.5 and 1. The AUC value cannot be less than 0.5. The drug-disease pairs are randomly removed from the interaction matrix **Y** before running cross validation. This method is called CV-p (Cross Validation pairs), and its purpose is to increase the difficulty of the prediction, thereby enabling a more complete assessment of the ability to predict new drugs. In addition, cross validation is performed on the training set to establish the parameters *λ*_*l*_, *λ*_*d*_ and *λ*_*t*_. Grid search is used to find the best parameter from the values: *λ*_*l*_ ∈ {2^−2^, 2^−1^, 2^0^, 2^1^}, *λ*_*d*_/*λ*_*t*_ ∈ {0, 10^−4^, 10^−3^, 10^−2^, 10^−1^}.

#### Prediction of the interaction under CV-p

Table 2 lists the experimental results of CV-p. The average of the AUC values of the ten cross validation results are taken as the final AUC score. Note that AUC is known to be insensitive to skewed class distributions [32]. The drug disease datasets are highly unbalanced in this study. In other words, there are more negative factors than positive factors. Therefore, the AUC value is a more appropriate measure to evaluate different methods. Table 2 shows the AUC values for different methods, and the best AUC value in each column is shown in bold. Standard deviations are shown in parentheses.

**Table 2:** AUC Results of Cross Validation Experiments

Methods	Cdataset	Fdataset
DrugNet	0.804 (0.001)	0.778(0.001)
KBMF	0.928(0.004)	0.915(0.003)
HGBI	0.858(0.014)	0.829(0.012)
MBiRw	0.933(0.003)	0.917(0.001)
DRRS	0.947(0.002)	0.930(0.001)
DNL_2,1_-CMF	0.951(0.001)	0.940(0.001)

As shown in Table 2, our proposed method, DNL_2,1_-CMF, achieves an AUC of 0.951 on the Cdataset, which is 0.4% higher than DRRS, with an AUC of 0.947. The AUC value of the DrugNet method is the lowest, and our method is 14.7% higher than this value. In addition, our approach also achieves the best results for the Fdataset. Our method achieves an AUC of 0.94, which is 1% higher than DRRS, with an AUC of 0.93. Additionally, the AUC value of the DrugNet method is the lowest, and our method is 16.2% higher than this value. Therefore, our proposed method is better than other existing methods.

In summary, the advantage of our method lies in the introduction of GIP and L_2,1_-norm. GIP can obtain network information on drugs and diseases. L_2,1_-norm can remove undesired drug disease pairs, thus improving prediction accuracy. Some of the previous methods only considered a single drug similarity and a single disease similarity and did not consider their network information. Therefore, our method can achieve better AUC values.

#### Sensitivity analysis from WKNKN

**Fig. 1:**
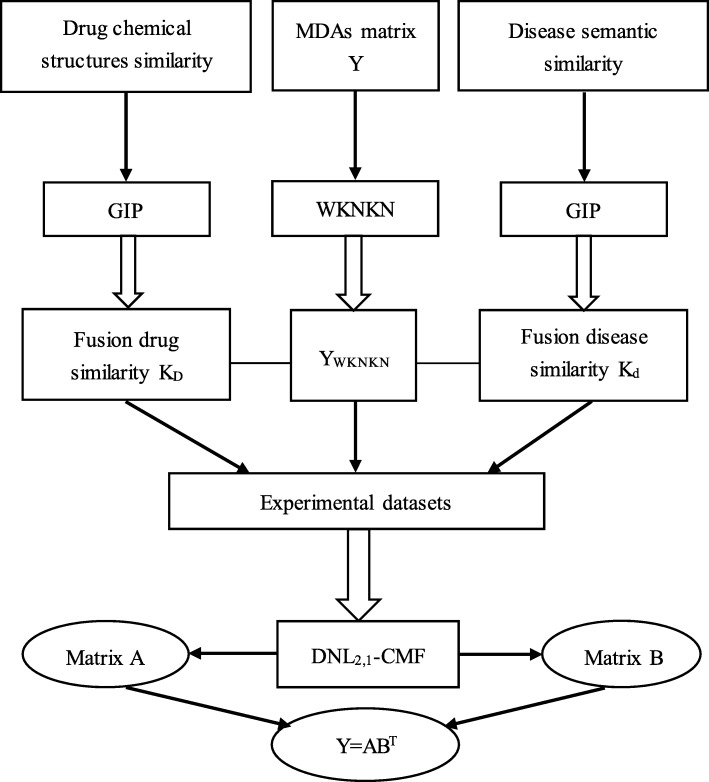
The flow chart from the original datasets to the final predicted score matrix

**Fig. 2:**
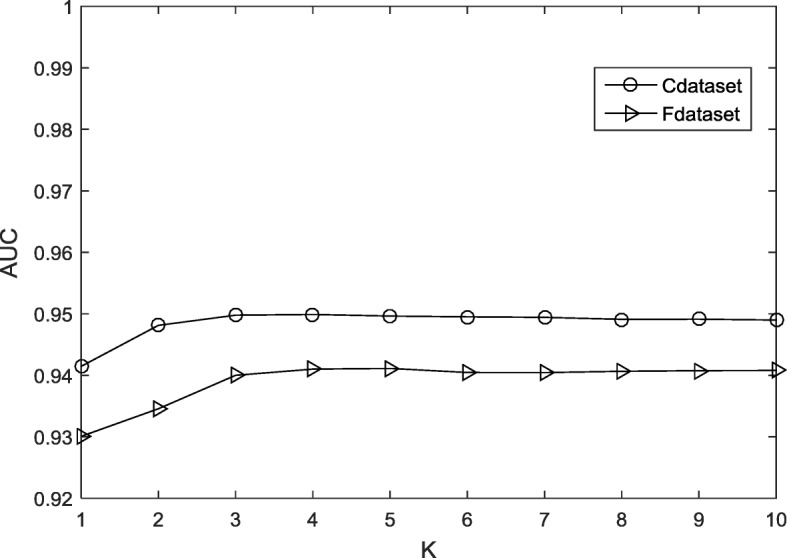
Sensitivity analysis for K under CV-p

As mentioned earlier in this paper, because there are some missing unknown interactions in the drug disease interaction matrix **Y**, a pre-processing method is used to minimize the error. The parameters *K* and *p* are fixed. *K* is the number of nearest known neighbours. *p* is a decay term where *p* ≤ 1, and WKNKN is used before running DNL_2,1_-CMF. When *K* = 5, *p* = 0.7, the AUC value approaches stability. The sensitivity analysis of these two parameters is shown in Figs. 1 and 2, respectively.

## Discussion

### Case study

In this subsection, a simulation experiment was conducted. Our method was used to predict the correct drugs in an unknown situation. Therefore, an unknown situation was created by removing some of the DDIs. **Y** was decomposed into two matrices, **A** and **B**, thus the product of these two matrices was used as the final prediction matrix. In this prediction matrix, all elements were no longer 0 and 1. Instead, all elements were close to 0 or 1. Therefore, we compared the elements in **Y** to determine the final prediction.

On the Cdataset, the seven pairs of interactions related to the drug zoledronic acid (KEGG ID: D01968) were completely removed. The drug was used to prevent skeletal fractures in patients with cancers such as multiple myeloma and prostate cancer. It can also be used to treat the hypercalcemia of malignancy and can be helpful for treating pain from bone metastases. A simulation was conducted to yield the prediction score matrix. Finally, the prediction score matrix counted whether those removed interactions were predicted. At the same time, the new interactions were counted. In other words, the disease most relevant to this drug was found. Among them, all known interactions and three novel interactions were successfully predicted. Table 3 lists the experimental results for the Cdataset. According to the level of relevance, these diseases were sorted from high to low. The known interactions are in bold. It is worth noting that according to our experimental analysis, the eighth disease, osteoporosis, had the strongest interaction with zoledronic acid. More information about the drug is published in DrugBank database.

**Table 3:** Predicted Diseases for Zoledronic acid, Cdataset

Rank	Disease	Disease ID
1	IBMPFD1	D167320
2	MYELOMA, MULTIPLE	D254500
3	MISMATCH REPAIR CANCER SYNDROME	D276300
4	PAGET DISEASE OF BONE 2, EARLY-ONSET	D602080
5	HAJDU-CHENEY SYNDROME	D102500
6	HEREDITARY LEIOMYOMATOSIS AND RENAL CELL CANCER	D605839
7	HYPERCALCEMIA, INFANTILE	D143880
8	OSTEOPOROSIS	D166710
9	RENAL CELL CARCINOMA,NON-PAPILLARY	D144700
10	ACROOSTEOLYSIS	D102400

The complete interactions of the drug hyoscyamine (KEGG ID: D00147) were removed. The drug is mainly used to treat bladder spasm, peptic ulcer disease, diverticulitis, colic, irritable bowel syndrome, cystitis and pancreatitis. This drug is also used to treat certain heart diseases and to control the symptoms of Parkinson’s disease and rhinitis. Fourteen pairs of interactions were removed, and these interactions were still predicted by our method. At the same time, motion sickness was predicted to be related to this drug. More information about the drug is published in https://www.drugbank.ca/drugs/DB00424. Table 4 lists the experimental results.

**Table 4:** Predicted Diseases for Hyoscyamine, Cdataset

Rank	Disease	Disease ID
1	TREMOR, NYSTAGMUS, AND DUODENAL ULCER	D190310
2	PARKINSON DISEASE, LATE-ONSET	D168600
3	PARK11	D607688
4	PARKINSON DISEASE, MITOCHONDRIAL	D556500
5	PARK15	D260300
6	PARK3	D602404
7	PARK1	D168601
8	PARK8	D607060
9	PARK7	D606324
10	PARK2	D600116
11	ENTEROCOLITIS	D226150
12	HYPERHIDROSIS PALMARIS ET PLANTARIS	D144110
13	ACANTHOSIS NIGRICANS WITH MUSCLE CRAMPS AND ACRAL ENLARGEMENT	D200170
14	PELGER-HUET-LIKE ANOMALY AND EPISODIC FEVER WITH ABDOMINAL PAIN	D260570
15	MOTION SICKNESS	D158280

For the Fdataset, the interactions of the drug cisplatin and the drug dexamethasone were removed, and a simulation experiment was conducted. Table 5 lists the experimental results for cisplatin, and Table 6 lists the experimental results for dexamethasone.

**Table 5:** Predicted Diseases for Cisplatin, Fdataset

Rank	Disease	Disease ID
1	LYMPHOMA,HODGKIN,CLASSIC	D236000
2	BLADDER CANCER	D109800
3	MISMATCH REPAIR CANCER SYNDROME	D276300
4	OSTEOGENIC SARCOMA	D259500
5	SMALL CELL CANCER OF THE LUNG	D182280
6	MYELOMA,MULTIPLE	D254500
7	OESOPHAGEAL CANCER	D133239
8	RHABDOMYOSARCOMA 2	D268220
9	PROSTATE CANCER, HEREDITARY, 1	D601518
10	LUNG CANCER	D211980

**Table 6:** Predicted Diseases for Dexamethasone, Fdataset

Rank	Disease	Disease ID
1	OTITIS MEDIA, SUSCEPTIBILITY TO	D166760
2	DERMATOSIS PAPULOSA NIGRA	D125600
3	MISMATCH REPAIR CANCER SYNDROME	D276300
4	ENTEROPATHY, FAMILIAL, WITH VILLOUS OEDEMA AND IMMUNOGLOBULIN G2 DEFICIENCY	D600351
5	THROMBOCYTOPENIC PURPURA, AUTOIMMUNE	D188030
6	HYPERTHERMIA, CUTANEOUS, WITH HEADACHES AND NAUSEA	D145590
7	GREENBERG DYSPLASIA	D215140
8	GROWTH RETARDATION, SMALL AND PUFFY HANDS AND FEET, AND ECZEMA	D233810
9	ASTHMA, NASAL POLYPS, AND ASPIRIN INTOLERANCE	D208550
10	MYCOSIS FUNGOIDES	D254400
11	DOHLE BODIES AND LEUKAEMIA	D223350
12	ATAXIA, EARLY-ONSET, WITH OCULOMOTOR APRAXIA AND HYPOALBUMINEMIA	D208920
13	ANAEMIA, AUTOIMMUNE HAEMOLYTIC	D205700
14	ADIE PUPIL	D103100
15	ENDOMETRIOSIS, SUSCEPTIBILITY TO, 1	D131200

For cisplatin (KEGG ID: D00275), nine interactions were removed. Six known interactions and three novel interactions were successfully predicted. The known interactions are shown in bold. More information about cisplatin is published at https://www.drugbank.ca/drugs/DB00515. For dexamethasone (KEGG ID: D00292), sixteen interactions were removed. Eleven known interactions and four novel interactions were successfully predicted. Moreover, endometriosis can be prevented by dexamethasone. In 2014, the ClinicalTrials.gov database was tested for this disease, and the reliability of this result has been confirmed by clinical trials. Sixty-four participants were used in the experiment. Detailed experimental results can be found at https://clinicaltrials.gov/ct2/show/study/NCT02056717. Diseases ranked 12, 13, and 14 were not confirmed by ClinicalTrials.gov for treatment with dexamethasone.

According to the above simulation results, our method has good performance for different datasets. According to Table 3 to Table 6, it can be concluded that the advantages of the L_2,1_-norm are increasing the disease matrix sparsity and discarding unwanted disease pairs. This advantage is reflected in the fact that in a drug-disease pair, unwanted noise is removed by the L_2,1_-norm, so the vast majority of known DDIs that have been removed are successfully predicted. Therefore, the addition of GIP kernels and L_2,1_-norm achieved better results than other advanced methods.

## Conclusions

In this paper, an effective matrix factorization model is proposed. L_2,1_-norm and GIP kernel are applied in this model. Moreover, the GIP kernel provides more network information for predicting novel DDIs. AUC is used to evaluate the indicators and our method achieves excellent results, so our method is feasible.

It is worth noting that the pre-processing method WKNKN plays an important role in prediction because there are many missing unknown interactions that are addressed by this pre-processing method. This is helpful for the final experimental results. However, the datasets used in this paper still have some limitations. For example, disease-disease similarity, sequence similarity and GO similarity are not considered. We will collect more similarity information in future work.

In the future, more datasets will be available, and more novel DDIs will be predicted. Of course, we will continue to employ more machine learning methods or deep learning methods to solve drug development problems.

## Methods

### Problem formalization

Formally, the known interactions **Y**(*D*(*i*), *d*(*j*)) of drug *D*(*i*) associated with disease *d*(*j*) are considered to be a matrix factorization model. The input matrix **Y** is decomposed into two low rank matrices **A** and **B**. These two matrices retain the features of the original matrix. Then, the two matrices are optimized through constraints. Finally, the specific matrices of **A** and **B** are obtained. Our mission is to rank all of the drug-disease pairs **Y**(*D*(*i*), *d*(*j*)). The most likely interaction pairs have the highest ranking.

### Gaussian interaction profile kernel

The method is based on the assumption that diseases that interact with DDIs networks and unrelated drugs in drug-disease networks may show similar interactions with new diseases. *D*(*i*) and *D*(*j*) represent two drugs, *d*(*i*) and *d*(*j*) represent two diseases. Their network similarity calculations can be written as:

3 $$ {GIP}_{Drug}\left({D}_{i,}{D}_j\right)=\exp \left(-\gamma {\left\Vert \mathbf{Y}\left({D}_i\right)-\mathbf{Y}\left({D}_j\right)\right\Vert}^2\right), $$

4 $$ {GIP}_{disease}\left({d}_{i,}{d}_j\right)=\exp \left(-\gamma {\left\Vert \mathbf{Y}\left({d}_i\right)-\mathbf{Y}\left({d}_j\right)\right\Vert}^2\right), $$

where *γ* is a parameter, which is used to adjust the bandwidth of the kernel. In addition, **Y**(*D*_*i*_) and **Y**(*D*_*j*_) are the interaction profiles of *D*_*i*_ and *D*_*j*_. Similarly, **Y**(*d*_*i*_) and **Y**(*d*_*j*_) are the interaction profiles of *d*_*i*_ and *d*_*j*_. Then, the two network similarity matrices can be combined with **S**_*D*_ and **S**_*d*_ to be written as:

5 $$ {\mathbf{K}}_{\mathrm{D}}=\alpha {\mathbf{S}}_{\mathrm{D}}+\left(1-\alpha \right){GIP}_D, $$

6 $$ {\mathbf{K}}_{\mathrm{d}}=\alpha {\mathbf{S}}_{\mathrm{d}}+\left(1-\alpha \right){GIP}_d, $$

where *α* ∈ [0, 1] is an adjustable parameter. **K**_D_ is a drug kernel, which represents a linear combination of the drug chemical similarity matrix **S**_D_ and the drug network similarity matrix *GIP*_*D*_. **K**_d_ is a disease kernel, which represents a linear combination of the disease semantic similarity matrix **S**_d_ and the disease network similarity matrix *GIP*_*d*_. Thus, the network information is applied to the prediction of DDIs and performed well in yielding results.

### Dual-network L_2,1_-collaborative matrix factorization (DNL_2,1_-CMF)

The traditional collaborative matrix factorization (CMF) uses collaborative filtering to predict novel interactions [25]. The objective function of CMF is given as follows:

7 $$ {\min}_{\mathbf{A},\mathbf{B}}={\left\Vert \mathbf{Y}-{\mathbf{A}\mathbf{B}}^{\mathrm{T}}\right\Vert}_F^2+{\lambda}_l\left({\left\Vert \mathbf{A}\right\Vert}_F^2+{\left\Vert \mathbf{B}\right\Vert}_F^2\right)+{\lambda}_d{\left\Vert {\mathbf{S}}_{\mathrm{D}}-{\mathbf{A}\mathbf{A}}^{\mathrm{T}}\right\Vert}_F^2+{\lambda}_t{\left\Vert {\mathbf{S}}_{\mathrm{d}}-{\mathbf{BB}}^{\mathrm{T}}\right\Vert}_F^2, $$

where ‖⋅‖_*F*_ is the Frobenius norm and *λ*_*l*_, *λ*_*d*_ and *λ*_*t*_ are non-negative parameters.

CMF is an effective method for predicting DDIs. However, this method ignores the network information of drugs and diseases. This problem will reduce the accuracy of the CMF method in predicting novel DDIs.

In this study, an improved collaborative matrix factorization method is used to predict DDIs. The L_2,1_-norm is added to the collaborative matrix factorization method, and drug network information and disease network information are combined with this method. The interaction matrix **Y** is decomposed into two matrices **A** and **B**, where **AB**^T^ ≈ **Y**.The dual-network L_2,1_-collaborative matrix factorization (DNL_2,1_-CMF) method uses regularization terms to request that the potential feature vectors of similar drugs and similar diseases are similar, and the potential feature vectors of dissimilar drugs and dissimilar diseases are dissimilar [33], where **S**_D_ ≈ **AA**^T^ and **S**_d_ ≈ **BB**^T^. Considering that GIP explores kernel network information, the dual-network can be interpreted as a drug network and a disease network generated by GIP. Specifically, the interaction profiles can be generated from a drug-disease interaction network. For a classifier, the interaction profiles can be used as feature vectors [34]. Therefore, the kernel method is used, and the kernel can be constructed from the interaction profiles. In summary, because of these advantages, GIP can achieve better results. Therefore, the objective function of DNL_2,1_-CMF method can be written as

8 $$ {\displaystyle \begin{array}{l}{\min}_{\mathbf{A},\mathbf{B}}={\left\Vert \mathbf{Y}-{\mathbf{A}\mathbf{B}}^{\mathrm{T}}\right\Vert}_F^2+{\lambda}_l\left({\left\Vert \mathbf{A}\right\Vert}_F^2+{\left\Vert \mathbf{B}\right\Vert}_F^2\right)+{\lambda}_l{\left\Vert \mathbf{B}\right\Vert}_{2,1}\\ {}+{\lambda}_d{\left\Vert {\mathbf{K}}_{\mathrm{D}}-{\mathbf{A}\mathbf{A}}^{\mathrm{T}}\right\Vert}_F^2+{\lambda}_t{\left\Vert {\mathbf{K}}_{\mathrm{d}}-{\mathbf{BB}}^{\mathrm{T}}\right\Vert}_F^2,\end{array}} $$

where‖⋅‖_*F*_ is the Frobenius norm and *λ*_*l*_, *λ*_*d*_ and *λ*_*t*_ are non-negative parameters. The first term is an approximate model of the matrix **Y**, whose purpose is to search the latent feature matrices **A** and **B**. The Tikhonov regularization is used to minimizes the norms of **A**, **B** in the second term, whose purpose is to avoid overfitting. The L_2,1_-norm is applied in **B** in the third term. The purpose is to increase the sparsity of the disease matrix and discard unwanted disease pairs. For a detailed explanation, please refer to [2]. Based on a previous study [25], the effect of the last two regularization terms is to minimize the squared error between **S**_D_(**S**_d_) and **AA**^T^(**BB**^T^).

#### Initialization of **A** and **B**

**Fig. 3:**
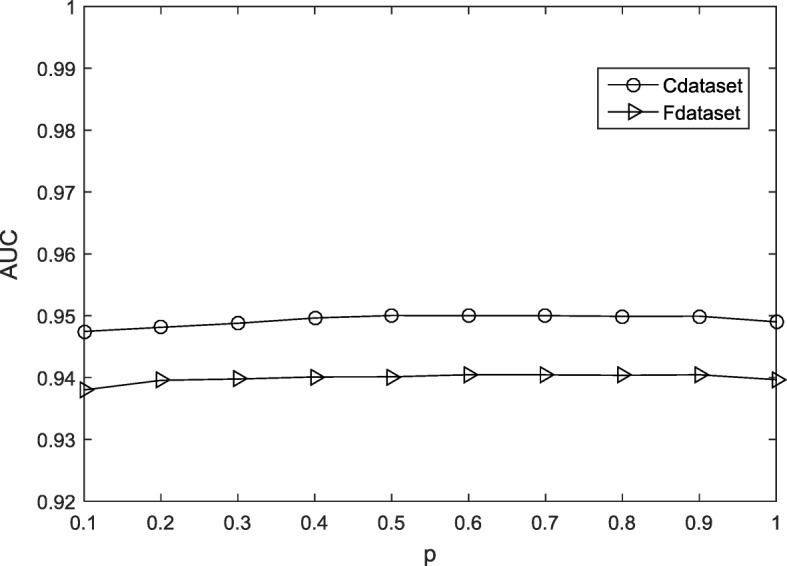
Sensitivity analysis for p under CV-p

For the input DDIs matrix **Y**, the singular value decomposition (SVD) method is used to obtain the initial value of matrix **A** and matrix **B**.

9 $$ \left[\mathbf{U},\mathbf{S},\mathbf{V}\right]=\mathrm{SVD}\left(\mathbf{Y},k\right),\mathbf{A}={\mathbf{US}}_{\mathrm{k}}^{1/2},\mathbf{B}={\mathbf{VS}}_{\mathrm{k}}^{1/2}, $$

where **S**_k_ is a diagonal matrix and contains the *k* largest singular values. In addition, the minimization of the objective function is used to predict the outcome of the interactions, but this could lead to unsatisfactory results. Many zeros have not been found, so the WKNKN pre-processing method is used to solve this problem. Figure 3 shows a specific prediction flow chart from the original datasets to the final predicted score matrix.

#### Optimization algorithm

In this study, the least squares method is used to update **A** and **B**. First, *L* is represented as the objection function of DNL_2,1_-CMF method. Then, *∂L*/*∂***A** and *∂L*/*∂***B** are set to be 0. According to the alternating least squares method, **A** and **B** are updated until convergence. It is worth noting that *λ*_*l*_, *λ*_*d*_ and *λ*_*t*_ are automatically determined by the cross validation on the training set to the optimal parameter values. Thus, the update rules are as follows:

10 $$ \mathbf{A}=\left(\mathbf{YB}+{\lambda}_d{\mathbf{K}}_{\mathrm{D}}\mathbf{A}\right){\left({\mathbf{B}}^{\mathrm{T}}\mathbf{B}+{\lambda}_l{\mathbf{I}}_{\mathrm{k}}+{\lambda}_d{\mathbf{AA}}^{\mathrm{T}}\right)}^{-1}, $$

11 $$ \mathbf{B}=\left({\mathbf{Y}}^{\mathrm{T}}\mathbf{A}+{\lambda}_t{\mathbf{K}}_{\mathrm{d}}\mathbf{B}\right){\left({\mathbf{A}}^{\mathrm{T}}\mathbf{A}+{\lambda}_l{\mathbf{I}}_{\mathrm{k}}+{\lambda}_t{\mathbf{B}}^{\mathrm{T}}\mathbf{B}+{\lambda}_l{\mathbf{DI}}_{\mathrm{k}}\right)}^{-1}. $$

According to formula (5) and formula (6), **K**_D_ can be represented by **S**_D_, and **K**_d_ can be represented by **S**_d_. These two complete updated rules can be written as:

12 $$ \mathbf{A}=\left(\mathbf{YB}+{\lambda}_d\left(\alpha {\mathbf{S}}_{\mathrm{D}}+\left(1-\alpha \right){GIP}_D\right)\mathbf{A}\right){\left({\mathbf{B}}^{\mathrm{T}}\mathbf{B}+{\lambda}_l{\mathbf{I}}_{\mathrm{k}}+{\lambda}_d{\mathbf{AA}}^{\mathrm{T}}\right)}^{-1}, $$

13 $$ \mathbf{B}=\left({\mathbf{Y}}^{\mathrm{T}}\mathbf{A}+{\lambda}_t\left(\alpha {\mathbf{S}}_{\mathrm{d}}+\left(1-\alpha \right){GIP}_d\right)\mathbf{B}\right){\left({\mathbf{A}}^{\mathrm{T}}\mathbf{A}+{\lambda}_l{\mathbf{I}}_{\mathrm{k}}+{\lambda}_t{\mathbf{B}}^{\mathrm{T}}\mathbf{B}+{\lambda}_l{\mathbf{DI}}_{\mathrm{k}}\right)}^{-1}, $$

where **D** is a diagonal matrix with the *i*-th diagonal element as *d*_*ii*_ = 1/2‖(**B**)^*i*^‖_2_. Therefore, the specific algorithm of DNL_2,1_-CMF is as follows:

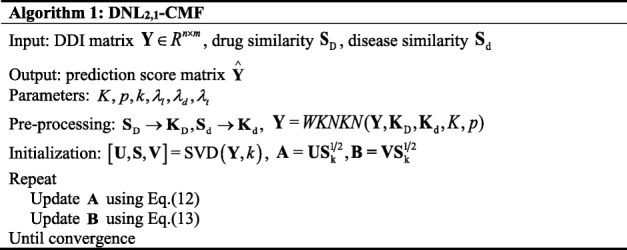



## Abbreviations

AUC: Area Under Curve
CMF: Collaborative Matrix Factorization
DDIs: Drug-Disease Interactions
DNL_2,1_-CMF: Dual-network L_2,1_-Collaborative Matrix Factorization
DRRS: Drug Repositioning Recommendation System
GIP: Gaussian Interaction Profile
KBMF2K: Kernel Bayesian Matrix Factorization
MBiRW: Measures and Bi-Random Walk
ROC: Receive Operating Characteristic
SVD: Singular Value Decomposition
SVT: Singular Value Thresholding
TPR: True Positive Rate; FPR: False Positive Rate
WKNKN: Weight K Nearest Known Neighbours
